# Association between fibromyalgia syndrome and peptic ulcer disease development

**DOI:** 10.1371/journal.pone.0175370

**Published:** 2017-04-06

**Authors:** Kevin A. Wang, Jia-Chi Wang, Cheng-Li Lin, Chun-Hung Tseng

**Affiliations:** 1 Division of General Surgery, Department of Surgery, Shin-Kong Memorial Hospital, Taipei, Taiwan; 2 School of Medicine, Fu Jen Catholic University, New Taipei City, Taiwan; 3 Department of Physical Medicine and Rehabilitation, National Yang-Ming University and Taipei Veterans General Hospital, Taipei, Taiwan; 4 Management Office for Health Data, China Medical University Hospital, Taichung, Taiwan; 5 College of Medicine, China Medical University, Taichung, Taiwan; 6 Graduate Institute of Clinical Medical Science and School of Medicine, College of Medicine, China Medical University, Taichung, Taiwan; 7 Department of Neurology, China Medical University Hospital, Taichung, Taiwan; University of Würzburg, GERMANY

## Abstract

**Purpose:**

The correlation of fibromyalgia syndrome (FMS) with peptic ulcer disease (PUD) is unclear. We therefore conducted a cohort study to investigate whether FMS is correlated with an increased risk of PUD.

**Methods:**

In this study, we established an FMS cohort comprising 26068 patients aged more than 20 years who were diagnosed with FMS from 2000 to 2011. Furthermore, we established a control cohort by randomly choosing 104269 people without FMS who were matched to the FMS patients by gender, age, and index year. All patients were free of PUD at the baseline. Cox proportional hazard regressions were performed to compute the hazard ratio of PUD after adjustment for demographic characteristics and comorbidities.

**Results:**

The prevalence of comorbidities was significantly higher in the FMS patients than in the controls. The incidence of PUD was 29.8 and 19.4 per 1000 person-years in the FMS and control cohorts, respectively. In addition, the FMS cohort exhibited a 1.40-fold higher risk of PUD (95% confidence interval = 1.35–1.45) compared with the control cohort. After control for confounding factors, the medications (selective serotonin reuptake inhibitors, serotonin–norepinephrine reuptake inhibitors, and antidepressants) taken by the FMS patients did not increase the risk of PUD.

**Conclusion:**

FMS patients exhibit a higher risk of PUD than that of patients without FMS.

## Introduction

Currently, fibromyalgia syndrome (FMS) is a complex condition affecting patients and can represent a diagnostic challenge for physicians. It is characterized as a pain processing disorder with several distinct secondary symptoms and is associated with low quality of life. [1–4] With the multitude of conditions contributing to FMS development, the exact cause of the disorder is unclear. However, it has been hypothesized that FMS is caused by an extensive list of factors, ranging from persistent inflammation and immunologic and muscular abnormalities to triggering [5] and maintenance factors. [6–9]

Approximately 50% of FMS patients often exhibit other illnesses, such as gastroesophageal reflux disease (GERD), irritable bowel syndrome, and other gastrointestinal disorders. [10–12] Among these illnesses, food sensitivities are an essential determinant of inflammation that might be associated to FMS pain. This pain and inflammation can be provoked by particular foods, such as preservatives, eggs, and gluten; however, the food causing FMS symptoms differs from person to person. Until now, few studies have demonstrated which specific foods are connected to FMS pain. [13–15] Moreover, recent studies have revealed that the severity of small intestinal bacterial outgrowth (SIBO) is correlated with FMS patients’ level of pain, indicating the significance of SIBO in FMS. [16,17] Furthermore, some researchers believe that FMS and gastrointestinal disorders occur in conjunction because their drivers—inflammation in the brain and gut or bacterial outgrowth in the intestines—are similar. [18]

The *Helicobacter pylori* bacterium is typically the causative agent of peptic ulcers, which are sores in the gastric lining, esophagus, or duodenum. These ulcers can also be attributed to the consistent use of nonsteroidal anti-inflammatory drugs (NSAIDs). Various classes of drugs, which often include NSAIDs, are utilized for treating FMS. However, despite their widespread use, results have shown their ineffectiveness in relieving FMS pain. [19] Therefore, physicians currently prescribe drugs that affect the central nervous system, [19,20] targeting the origins of pain reception and slowly eliminating the use of NSAIDs in FMS treatment.

Some physicians believe that stress [21] may play a role in the activity of the gut through its effect on hormones and nerves [22,23], although the link is yet to be confirmed. To the best of our knowledge, the epidemiological evidence for the association of FMS with the risk of PUD is still insufficient. Therefore, in this population-based study, we investigated the relationship between FMS and PUD development.

## Methods

### Data source

The National Health Insurance (NHI) program in Taiwan is a single-payer universal insurance program implemented on March 1, 1995, and the NHI program covers approximately 99% of the Taiwanese population. [24] The National Health Insurance Administration has authorized the National Health Research Institutes (NHRI) to create an encrypted, secondary database—the National Health Insurance Research Database (NHIRD)—for research purposes. In this study, we analyzed the Longitudinal Health Insurance Database 2000 (LHID2000), which constitutes a subdataset of the NHIRD. The details of the LHID2000 are provided in previous studies. [25,26] Diagnoses were classified according to the International Classification of Diseases, Ninth Revision, Clinical Modification (ICD-9-CM) codes.

### Data availability statement

The dataset used in this study is held by the Taiwan Ministry of Health and Welfare (MOHW). The Ministry of Health and Welfare must approve our application to access this data. Any researcher interested in accessing this dataset can submit an application form to the Ministry of Health and Welfare requesting access. Please contact the staff of MOHW (Email: stcarolwu@mohw.gov.tw) for further assistance. Taiwan Ministry of Health and Welfare Address: No.488, Sec. 6, Zhongxiao E. Rd., Nangang Dist., Taipei City 115, Taiwan (R.O.C.). Phone: +886-2-8590-6848. All relevant data are within the paper.

### Ethics statement

The NHIRD encrypts patient personal information to ensure patient privacy, and researchers are provided with anonymous identification numbers associated with the relevant claims information, including sex, date of birth, medical services received, and prescriptions. Therefore, patient consent is not required to access the NHIRD. This study was approved to fulfill the condition for exemption by the Institutional Review Board (IRB) of China Medical University (CMUH104-REC2-115-CR1). The IRB also specifically waived the consent requirement.

### Study population

This study was assessed on the risk of PUD between the individual with and without FMS. FMS, characterized by widespread musculoskeletal pain and multiple tender points, was diagnosed by rheumatologists, neurologists, psychologists, physiatrists, and pain specialists with clinical accuracy, according to the American College of Rheumatology Criteria for the Classification of Fibromyalgia. [27] Patients aged ≥20 years who were diagnosed with FMS (ICD-9-CM code 729.1) more than three times within 3 months were included in the FMS cohort. The index date was defined as the first diagnosis date of FMS. To establish a control cohort, patients without FMS were randomly selected and matched to the FMS patients at a 4:1 ratio by age group (every 5-year span), sex, and index date. The exclusion criteria were a history of PUD (ICD-9-CM codes 531–533) before the index date and missing information.

### Outcome

The outcome of interest was a new diagnosis of PUD from 2000 to 2011. Both the FMS and control cohorts were monitored until diagnosis of PUD or until the patients were censored because of withdrawal from the NHI program or the end of 2011.

### Comorbidities and medications

To evaluate the potential risk and to control for confounding factors, we included the comorbidities and medications of each patient, namely hyperlipidemia (ICD-9-CM code 272), diabetes (ICD-9-CM code 250), liver cirrhosis (ICD-9-CM codes 571.2, 571.5, and 571.6), alcohol-related illness (ICD-9-CM codes 291, 303, 305, 571.0, 571.1, 571.2, 571.3, 790.3, A215, and V11.3), hypertension (ICD-9-CM codes 401–405), depression (ICD-9-CM codes 296.2, 296.3, 300.4, and 311), anxiety (ICD-9-CM code 300.0), sleep disorder (ICD-9-CM codes 307.4 and 780.5), stroke (ICD-9-CM codes 430–438), *H*. *pylori* infection (ICD-9-CM code 041.86), GERD (ICD-9-CM codes 530.11 and 530.81), and proton pump inhibitor (PPI) and NSAID use. Furthermore, we assessed whether FMS medications, including amitriptyline, fluoxetine, duloxetine, milnacipran, meclobemide, tropisetron, pramipexole, and pregabalin, play a role in PUD outcomes.

### Statistical analysis

The chi-square test was used for analyzing categorical variables, and the Student’s *t* test was used for analyzing continuous variables. The cumulative incidence of PUD in the FMS and control cohorts was explored using the Kaplan–Meier method, and the differences were determined using log-rank tests. The incidence density rates were calculated by dividing the number of PUD events by the total follow-up years (per 1st000 person-years). The incidence density rates of PUD for each risk factor and stratified by age, sex, comorbidity, and medications in the both cohorts were calculated. Univariable and multivariable Cox proportional hazard regression models were used to determine the risk factors for PUD, denoted as a hazard ratio (HR) with a 95% confidence interval (CI). Stratified analysis of PUD risk by age, sex, comorbidities and medications was also estimated by the Cox models. The multivariable models included all statistically significant risk factors identified in the univariable model. Data management and analyses were performed using SAS 9.4 software (SAS Institute, Cary, NC, USA). A two-tailed *P* value < 0.05 was considered significant.

## Results

**Fig 1:**
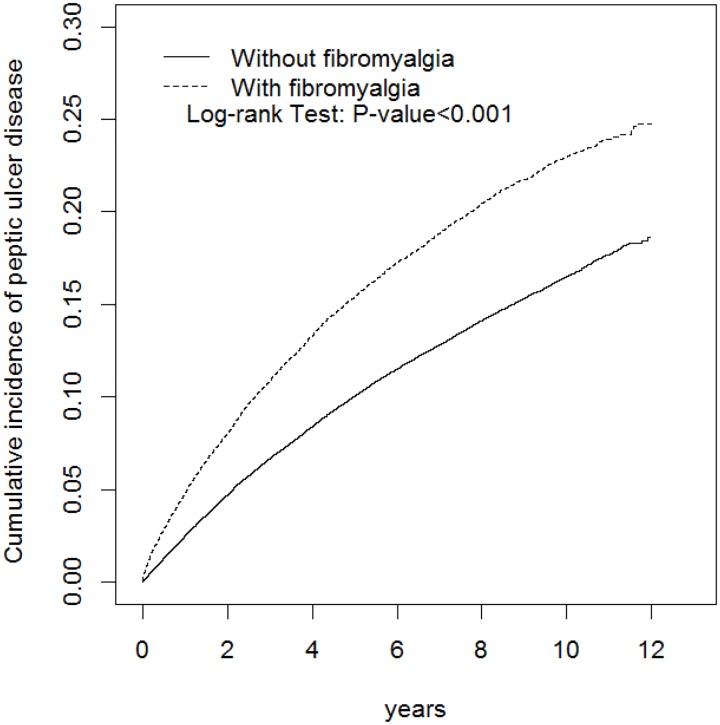
Comparison of cumulative incidence of peptic ulcer disease in patients with (dashed line) and those without (solid line) fibromyalgia syndrome.

A total of 26068 FMS patients and 104269 controls were included in this study (Table 1). Most patients were aged ≤49 years (53.8%) and were women (54.6%). The mean ages of the FMS and control cohorts were 49.5 ± 16.0 and 49.0 ± 16.3 years, respectively. The comorbidities of hyperlipidemia, diabetes, liver cirrhosis, alcohol-related illness, hypertension, depression, anxiety, sleep disorder, stroke, and GERD and NSAID use were more prevalent in the FMS cohort than in the control cohort. The average follow-up durations were 5.59 and 5.87 years in the FMS and control FMS cohorts, respectively. As shown in Fig 1, the cumulative incidence of PUD was higher in the FMS cohort than in the control cohort (log-rank test *P* < 0.001).

**Table 1:** Demographic characteristics and comorbidities in patients with and without fibromyalgia syndrome.

	Non-FM cohort	FM cohort	
Variable	N = 104269	N = 26068	*p*-value
**Age, year**			0.99
≤ 49	56100(53.8)	14025(53.8)	
50–64	28020(26.9)	7005(26.9)	
65+	20149(19.3)	5038(19.3)	
Mean±SD^†^	49.0(16.3)	49.5(16.0)	<0.001
**Sex**			0.99
Female	56972(54.6)	14243(54.6)	
Male	47297(45.4)	11825(45.4)	
**Comorbidity**			
Hyperlipidemia	14314(13.7)	4900(18.8)	<0.001
Diabetes	6838(6.56)	2159(8.28)	<0.001
Liver cirrhosis	468(0.45)	150(0.58)	0.008
Alcohol-related illness	2148(2.06)	714(2.74)	<0.001
Hypertension	24322(23.3)	7673(29.4)	<0.001
Depression	2561(2.46)	1064(4.08)	<0.001
Anxiety	3695(3.54)	1624(6.23)	<0.001
Sleep disorder	12290(11.8)	5072(19.5)	<0.001
Stroke	2633(2.53)	874(3.35)	<0.001
Gastroesophageal reflux disorder	439(0.42)	168(0.64)	<0.001
H. pylori infection	42(0.04)	11(0.04)	0.89
**Medication**			
NSAID	42400(40.7)	14738(56.5)	<0.001

Chi-square test;

^†^: *t* test

NSAID, nonsteroidal anti-inflammatory drug

The incidence density rate of PUD was 29.8 per 1000 person-years in the FMS cohort, which was significantly higher than that in the control cohort (19.4 per 1000 person-years; Table 2). The FMS cohort exhibited a 1.40-fold higher risk of PUD compared with the control cohort (95% CI = 1.35–1.45). Compared with patients aged ≤49 years, the risk of PUD was 1.58- and 1.96-fold higher in those aged 50–64 years and ≥65 years (95% CI = 1.52–1.64 and 1.88–2.05, respectively). The risk of PUD was higher in patients with the comorbidities of hyperlipidemia [adjusted HR (aHR) = 1.22, 95% CI = 1.17–1.27], liver cirrhosis (aHR = 1.76, 95% CI = 1.48–2.09), hypertension (aHR = 1.24, 95% CI = 1.19–1.29), depression (aHR = 1.19, 95% CI = 1.09–1.29), anxiety (aHR = 1.15, 95% CI = 1.10–1.20), and sleep disorder (aHR = 1.15, 95% CI = 1.10–1.20). The aHR of PUD development was high in patients taking NSAIDs (aHR = 1.28, 95% CI = 1.24–1.33).

**Table 2:** Incidence and risk factors for peptic ulcer disease.

Variable	Event	PY	Rate^#^	Crude HR (95% CI)	Adjusted HR^†^ (95% CI)
**Fibromyalgia**					
Non-FM cohort	11879	612540	19.4	1.00	1.00
FM cohort	4337	145634	29.8	1.53(1.48, 1.58)***	1.40(1.35, 1.45)***
**Age, year**					
≤ 49	6258	435894	14.4	1.00	1.00
50–64	5309	198789	26.7	1.84(1.77, 1.91)***	1.58(1.52, 1.64)***
65+	4649	123491	37.7	2.54(2.44, 2.63)***	1.96(1.88, 2.05)***
**Sex**					
Female	9287	426012	21.8	1.00	1.00
Male	6929	332162	20.9	1.06(1.02, 1.09)***	1.02(0.98, 1.05)
**Comorbidity**					
**Hyperlipidemia**					
No	12600	659721	19.1	1.00	1.00
Yes	3616	98453	36.7	1.88(1.81, 1.95)***	1.22(1.17, 1.27)***
**Diabetes**					
No	14707	715952	20.5	1.00	1.00
Yes	1509	42222	35.7	1.68(1.59, 1.77)***	1.00(0.94, 1.06)
**Liver cirrhosis**					
No	16088	755855	21.3	1.00	1.00
Yes	128	2319	55.2	2.43(2.04, 2.89)***	1.76(1.48, 2.09)***
**Alcohol-related illness**					
No	15895	746747	21.3	1.00	1.00
Yes	321	11427	28.1	1.24(1.11, 1.38)***	1.06(0.95, 1.19)
**Hypertension**					
No	10280	592447	17.4	1.00	1.00
Yes	5936	165727	35.8	2.02(1.96, 2.09)***	1.24(1.19, 1.29)***
**Depression**					
No	15610	741182	21.1	1.00	1.00
Yes	606	16992	35.7	1.63(1.51, 1.77)***	1.19(1.09, 1.29)***
**Anxiety**					
No	15325	734729	20.9	1.00	1.00
Yes	891	23445	38.0	1.73(1.62, 1.85)***	1.15(1.10, 1.20)***
**Sleep disorder**					
No	13513	676459	20.0	1.00	1.00
Yes	2703	81716	33.1	1.59(1.52, 1.65)***	1.15(1.10, 1.20)***
**Stroke**					
No	15665	743843	21.1	1.00	1.00
Yes	551	14331	38.5	1.72(1.58, 1.87)***	0.97(0.89, 1.06)
**Gastroesophageal reflux disorder**					
No	16157	756844	21.4	1.00	1.00
Yes	59	1330	44.4	1.75(1.35, 2.25)***	1.16(0.90, 1.51)
**H. pylori infection**					
No	16215	758066	21.4	1.00	1.00
Yes	1	108	9.25	0.36(0.05, 2.53)	-
**Medication**					
**NSAID**					
No	7853	472003	16.6	1.00	1.00
Yes	8363	286171	29.2	1.69(1.64, 1.74)***	1.28(1.24, 1.33)***

PY, person-years;

Rate^#^, incidence rate, per 1000 person-years; crude HR, relative hazard ratio;

adjusted HR^†^: multivariable analysis including age; sex; comorbidities of hyperlipidemia, diabetes, liver cirrhosis, alcohol-related illness, hypertension, depression, anxiety, sleep disorder, stroke, and gastroesophageal reflux disorder; and NSAID use.

***P < 0.001

Table 3 shows a comparison of PUD incidence and the Cox model-measured hazards ratio between the patients with FMS and those without FMS after stratification by age, sex, comorbidity, and medications. Regardless of stratification, the risk of PUD was higher in the FMS patients than in the controls.

**Table 3:** Incidence of peptic ulcer disease by age, sex, comorbidity, and medications and Cox model-measured hazard ratio for patients with fibromyalgia syndrome compared those without fibromyalgia syndrome.

	Non-FM cohort			FM cohort				
Variables	Event	PY	Rate^#^	Event	PY	Rate^#^	Crude HR (95% CI)	Adjusted HR^†^ (95% CI)
**Age, years**								
≤ 49	4423	351605	12.6	1835	84290	21.8	1.72(1.63, 1.82)***	1.52(1.44, 1.61)***
50–64	3947	161146	24.5	1362	37643	36.2	1.47(1.38, 1.56)***	1.32(1.24, 1.40)***
65+	3509	99789	35.2	1140	23702	48.1	1.36(1.27, 1.45)***	1.3091.22, 1.39)***
**Sex**								
Female	6825	344075	19.8	2462	81937	30.1	1.51(1.44, 1.58)***	1.37(1.31, 1.44)***
Male	5054	268465	18.8	1875	63697	29.4	1.56(1.48, 1.64)***	1.45(1.37, 1.53)***
**Comorbidity**^‡^								
No	5608	408359	13.7	1621	77476	20.9	1.5291.44, 1.61)***	1.48(1.40, 1.56)***
Yes	6271	204181	30.7	2716	68158	39.9	1.30(1.24, 1.36)***	1.33(1.27, 1.39)***
**Medication**								
**NSAID**								
No	6195	402114	15.4	1658	69890	23.7	1.53(1.45, 1.62)***	1.50(1.42, 1.59)***
Yes	5684	210426	27.0	2679	75745	35.4	1.32(1.26, 1.39)***	1.33(1.27, 1.39)***

PY, person-years;

Rate^#^, incidence rate, per 1000 person-years; crude HR, relative hazard ratio;

adjusted HR^†^: multivariable analysis including age; sex; comorbidities of hyperlipidemia, diabetes, liver cirrhosis, alcohol-related illness, hypertension, depression, anxiety, sleep disorder, and stroke; and NSAID use.

Comorbidity^‡^: Patients with any one of the comorbidities of hyperlipidemia, diabetes, liver cirrhosis, alcohol-related illness, hypertension, depression, anxiety, sleep disorder, and stroke.

***P < 0.001

Table 4 displays the results of an analysis of the effects of FMS medications on the risk of PUD compared with the control cohort. FMS patients who did not receive medications exhibited a significantly 1.48-fold higher risk of PUD (95% CI = 1.42–1.53) compared with the controls. Patients receiving meclobemide, fluoxetine, tropisetron, duloxetine, or milnacipran exhibited a significantly 1.64-fold higher risk of PUD (95% CI = 1.28–2.10) compared with the controls. FMS patients who received pregabalin, amitriptyline, or pramipexole exhibited a significantly 1.55-fold higher risk of PUD (95% CI = 1.22–1.97) compared with the controls.

**Table 4:** Incidence and hazard ratio of peptic ulcer disease among fibromyalgia syndrome patients with and without treatment and compared with controls.

Variables	N	Event	PY	Rate^#^	Crude HR (95% CI)	Adjusted HR^†^ (95% CI)
**Non- fibromyalgia controls**	104269	11879	612540	19.4	1(Reference)	1(Reference)
**Fibromyalgia**						
Without medications	24201	4118	133790	30.8	1.58(1.52, 1.63)***	1.48(1.42, 1.53)***
Treatment with pregabalin, amitriptyline, pramipexole	265	68	1861	36.5	1.95(1.54, 2.48)***	1.55(1.22, 1.97)***
Treatment with meclobemide, fluoxetine, tropisetron, duloxetine, or milnacipran	261	63	1776	35.5	1.87(1.46, 2.39)**	1.64(1.28, 2.10)***

PY, person-years;

Rate^#^, incidence rate, per 1000 person-years; crude HR, relative hazard ratio;

adjusted HR^†^: hazard ratio from multivariable analysis including age; sex; comorbidities of hyperlipidemia, diabetes, liver cirrhosis, alcohol-related illness, hypertension, depression, anxiety, sleep disorder, and stroke; and NSAID use.

**P < 0.01,

***P < 0.001

## Discussion

This is the first study that showed the long-term risk of PUD in FMS patients by using a population-based database. Through the primary findings, our hypothesis that FMS patients have an elevated risk of PUD is proven true. At the end of the follow-up period, the cumulative frequency of PUD was higher in the FMS cohort than in the control cohort (Fig 1). The incidence density rates of PUD were 29.8 and 19.4 per 1000 person-years in the FMS and control cohorts, respectively. Moreover, the FMS cohort exhibited a 1.40-fold higher risk of PUD (95% CI = 1.35–1.45) compared with the control cohort (Table 2).

The prevalence rate of comorbidities was also compared between the FMS and control cohorts, as shown in Table 1. Comorbidities such as hyperlipidemia, diabetes, liver cirrhosis, hypertension, depression, anxiety, sleep disorder, stroke, and GERD and PPI and NSAID use were more common in the FMS cohort than in the control cohort. Although not a primary concern of the current study, certain illnesses are expected to be more prevalent in the FMS cohort. [2,28] Because FMS is a complex condition that is likely multifactorial, both internal and external factors may be triggers for PUD development. [28–32]

The mechanisms underlying the association of FMS with an increased risk of PUD are unclear. However, many patients reported an intestinal infection as the initial symptom of FMS; therefore, current and past infection with common intestinal pathogens (i.e., *H*. *pylori*) might induce PUD development in FMS patients. [16,17,33] In addition, gut infection, gut inflammation, medications, stress, and trauma can induce PUD development and damage the mucosal barrier of the gastrointestinal epithelium, [34–36] which allows unshielded molecules to enter the bloodstream (known as leaky gut syndrome). The leaky gut causes systemic inflammation and triggers immune responses, leading to a wide array of diseases, including FMS. [34–36]

The risk of PUD in FMS patients receiving medications and those not receiving medications was 1.59-fold (95% CI = 1.34–1.89) and 1.48-fold (95% CI = 1.42–1.53) higher than that in the controls. Drugs that affect serotonin levels (fluoxetine, tropisetron, duloxetine, meclobemide, and milnacipran) were shown to increase the risk of gastrointestinal problems by other investigators [37–41], but they did not exert more adverse effects than those of other medications (pregabalin, amitriptyline, and pramipexole) in our study (aHR = 1.64 and 1.55, respectively). Our findings show that the listed drugs may not be the cause of ulcers (Table 4).

Increasing evidence has shown that bioenergetics and mitochondrial function are impaired in FMS [42–44] and PUD [45–47] patients. Although it is unclear whether oxidative stress is a common pathway of PUD and FMS, recent studies have shown that oxidative stress can cause the pathophysiological mechanisms that culminate in the symptoms of PUD and FMS.

This study has some limitations. First, the data extracted from the NHIRD represent only the incidents at discharge; discrepancies between medical treatments and patient diagnoses cannot be directly verified. Second, our study did not assess the severity of FMS and PUD; therefore, we cannot for certain state how the FMS severity affects the subsequent risk of PUD. Moreover, the evidence in this study may be restricted to Taiwan, because it was obtained using the claims data in the NHIRD for feasibility and practicality. Finally, the patients’ diet, exposure to smoking and alcohol, and psychological factors are not available in the NHI dataset; therefore, these factors could not be estimated when determining the PUD risk.

## Conclusion

In this study, we demonstrated that FMS contributes to an elevated risk of PUD. FMS patients had a high prevalence of comorbidities, and the drugs identified that relieve psychosomatic symptoms in FMS did not increase the likelihood of ulcers. The mechanisms underlying the link between FMS and PUD are still unclear. Additional studies are required to clarify the underlying mechanisms.

## Supporting information

S1 STROBE Checklist Checklist of items that should be included in reports of observational studies. (DOC)

## Abbreviations

CI: confidence interval
FMS: fibromyalgia syndrome
GERD: gastroesophageal reflux disease
HR: hazard ratio
ICD-9-CM: International Classification of Diseases, Ninth Revision, Clinical Modification
LHID2000: Longitudinal Health Insurance Database 2000
NHIRD: National Health Insurance Research Database
NSAIDs: nonsteroidal anti-inflammatory drugs
PUD: peptic ulcer disease
SIBO: small intestinal bacterial outgrowth
